# Assessing the Impact of Ambidextrous Leadership on Nurses’ Presenteeism: A Latent Profile and Mediation Analysis Study

**DOI:** 10.1155/jonm/6624868

**Published:** 2026-02-27

**Authors:** Xiuran Zhou, Ying Yu, Xiaoying Yan

**Affiliations:** ^1^ School of Nursing, Guangdong Pharmaceutical University, Guangzhou, Guangdong, China, gdpu.edu.cn; ^2^ Guangzhou Municipal Health Commission, Guangzhou, Guangdong, China

**Keywords:** ambidextrous leadership, latent profile analysis, leader–member exchange, mediation analysis, nurses’presenteeism

## Abstract

**Background:**

The high incidence of nurse presenteeism significantly impact quality of care delivery. Leader–member exchange and clinical leadership have been identified as factors influencing nurses’ presenteeism. However, there are few studies investigating the relationship between these variables. This study aimed to investigate the heterogeneity of ambidextrous leadership and its relationships with nurses’ presenteeism using latent profile analysis, while also examining the mediating role of leader–member exchange.

**Methods:**

A multicenter cross‐sectional survey was conducted from December 2024 to January 2025, involving clinical nurses from three tertiary general hospitals in Guangzhou. Sociodemographic Characteristics Questionnaire, Ambidextrous Leadership Scale, Leader–Member Exchange Scale, and Stanford Presenteeism Scale were used. Latent profile analysis was performed using Mplus 8.3, and mediation effects were tested through PROCESS 4.2 in SPSS 27.0.

**Results:**

A total of 513 valid questionnaires were collected. Three ambidextrous leadership profiles were identified: ambidextrous lagging, vision‐oriented, and ambidextrous synergistic. These profiles showed significant differences in the leader–member exchange and presenteeism scores. Moreover, leader–member exchange significantly mediated the relationship between ambidextrous leadership and nurses’ presenteeism.

**Conclusions:**

Ambidextrous leadership is heterogeneous. The leader–member exchange mediates ambidextrous leadership and nurses’ presenteeism. Therefore, nursing managers should be flexible in changing their leadership styles to reduce nurses’ presenteeism.

**Implications for Nursing Management:**

There should be further intensive training for nursing managers to implement scientifically sound leadership functions, enhance clinical nurse–manager relationships, and increase nurses’ motivation, thereby reducing the incidence of presenteeism.

## 1. Introduction

Improving healthcare service quality remains a critical global challenge [1]. As the primary healthcare workforce providing frontline clinical services, nurses’ work engagement significantly influences both patient safety outcomes and care quality metrics [2]. However, recent epidemiological studies have revealed a concerning prevalence of presenteeism among nurses, where they are physically present at work but experience reduced productivity due to poor physical or mental health [3, 4].

Nursing has been identified as the occupational group with the highest prevalence of presenteeism [5]. A recent multinational meta‐analysis covering 14 countries revealed that 49.2% of nurses experience presenteeism [6], and this phenomenon was significantly worsened by the global nursing workforce crisis during the COVID‐19 pandemic [7, 8]. Empirical evidence indicates that nurses working with untreated health conditions demonstrate reduced clinical performance, such as impaired concentration and delayed response times. These manifestations lead to compromised care quality and increased patient safety risks, particularly in terms of medication administration errors and procedural compliance rates [9].

Leadership style has been identified as a critical determinant of nurses’ work attitudes and behavioral outcomes. Empirical evidence indicates that supportive leadership approaches significantly reduce presenteeism by improving psychological safety and optimizing resource allocation [10]. Conversely, authoritarian leadership styles, which are characterized by rigid control and limited autonomy, have been found to exacerbate occupational stress. This, in turn, leads to an increase in presenteeism rates [11]. Inclusive leadership models demonstrate positive correlations with workforce motivation and organizational efficiency through trust‐based supervisor–subordinate relationships [10]. Current research is mainly limited to the examination of singular leadership paradigms. This methodological limitation obscures potential interactive effects between different leadership dimensions and fails to account for situational moderators that may influence nursing outcomes. Accordingly, different leadership styles are required, and ambidextrous leadership offers a promising perspective to address this limitation.

Ambidextrous leadership refers to a leader’s capacity to achieve synergy and flexibly alternate between two contradictory leadership approaches in response to external contexts. The core concept of ambidextrous leadership originally originates from March’s [12] theory of organizational ambidexterity, which emphasizes a leader’s flexibility in transitioning between “exploration” and “exploitation” behaviors. Exploratory behavior emphasizes the discovery of novel ideas and encourages employees to challenge the status quo and experiment with innovative approaches. In contrast, exploitative behavior focuses on the comprehensive utilization of existing resources and efficient operations, frequently through the establishment of standardized processes and systems, along with enhanced monitoring and evaluation, thus ensuring overall efficiency. In existing research, scholars have integrated ambidextrous leadership behaviors from multiple perspectives. From a cognitive perspective [13], they integrated exploratory and exploitative leadership behaviors [14] and combined open and closed leadership. They argue that leaders need to balance the utilization of existing knowledge and experience with the exploration of new ideas and knowledge when addressing managerial issues. From a rights perspective [15], they integrated empowering and directive leadership, examining leadership effectiveness through the combination of varying degrees of power concentration and distribution. From a conventional perspective [16, 17], they integrated transformational and transactional leadership into a framework. Transformational leadership, which emphasizes vision, inspiration, and the encouragement of innovative thinking, serves as a strong predictor of exploratory leadership behaviors. This type of leadership motivates employees to think critically, explore new ideas, and adopt unconventional solutions, characteristics that closely align with an exploratory leadership approach [16]. In contrast, transactional leadership focuses on clarifying tasks through contractual agreements, monitoring performance to ensure procedural compliance, and managing by exception to correct deviations. These structured management methods are particularly suitable in exploitative contexts that emphasize stability and consistency, thereby effectively enhancing organizational operational efficiency [18]. Therefore, the ambidextrous leadership that combines transformational and transactional leadership serves as an effective pathway for organizations to achieve innovation and efficient operations in dynamic environments. Furthermore, the author Chebbi [19] indicated that ambidextrous leadership, composing transformational and transactional leadership, is particularly suitable for developing countries. This study, considering China’s developmental context, defines ambidextrous leadership as the combination of transformational and transactional leadership. Ambidextrous leadership, characterized by the integration of transformational and transactional leadership, has emerged as a crucial strategy for addressing the complexity of modern healthcare environments. Relevant research confirms that the synergy between transformational and transactional leadership is effective in improving nurses’ performance [20].

How then does ambidextrous leadership impact nurses’ presenteeism, and via what mechanisms? Studies suggest that psychological mechanisms can mediate the relationship between leadership and nurses’ presenteeism [10]. However, the underlying psychological mechanisms regarding this relationship have not yet been fully clarified. Since leadership can influence nurses’ psychological connection with their leaders and teams, thereby resulting in varying attitudes and behaviors [21]. Therefore, this study examines the relationship between ambidextrous leadership and nurses’ presenteeism from the social exchange perspective, with leader–member exchange (LMX) regarded as a crucial psychological mediator in this relationship.

The social exchange theory (SET) posits that interpersonal relationships are governed by the reciprocal exchange of resources, whereby individuals maintain relational equity by mutually providing benefits [22]. Based on this framework, the relationship between ambidextrous leadership and nurses’ presenteeism can be understood as an exchange dynamic. Combining transformational leadership, which emphasizes challenging the status quo and breaking existing frameworks, with transactional leadership, which emphasizes planning and assigning roles and tasks, creates synergistic effects. These effects contribute to a more effective mitigation of nurses’ presenteeism. In this interactive process, nurses reciprocate by decreasing their presenteeism in response to leadership behaviors.

LMX represents a type of supervisor–subordinate interaction based on the principle of reciprocity, emphasizing the establishment of trust between leaders and followers [23]. However, leaders do not treat all subordinates uniformly; instead, they develop relationships of varying quality with different followers. Grounded in SET, ambidextrous leadership can act as a significant facilitator of LMX, potentially exerting further influencing nurses’ attitudes and behaviors. The main reason is that the dual features of ambidextrous leadership, namely, task‐oriented and care‐oriented behaviors, can be regarded as significant investments made by leaders in nurses. Specifically, transformational leadership stimulates nurses’ intrinsic motivation, organizational identification, and psychological safety through articulating a vision, creating meaning, and focusing on their development [24]. In contrast, transactional leadership establishes an exchange relationship with the nurses based on the balance of rights and responsibilities through explicit goal‐setting, performance evaluation, and reward–punishment mechanisms [25]. Under the synergistic effect of these two leadership styles, high‐quality LMX relationships are more likely to be established between leaders and nurses.

High‐quality LMX implies greater trust, two‐way communication, resource support, and emotional identification. Such a relationship fosters a sense of mutual obligation and responsibility between leaders and nurses. When nurses perceive the “investment signals” conveyed by ambidextrous leadership, they interpret this support as a social favor. Driven by the norm of reciprocity, nurses are likely to enhance their awareness of organizational citizenship and sense of responsibility and exhibit positive behaviors, such as reducing presenteeism, to fulfill this trust‐based obligation. These positive organizational behaviors, in turn, reinforce leaders’ trust and support for nurses, thus forming a virtuous cycle.

This study utilizes latent profile analysis (LPA) to explore the combination patterns of transformational and transactional leadership. The reason is that traditional research often uses variable‐centered methods, treating transformational and transactional leadership as two independent dimensions to examine their independent or interactive effects on outcome variables. However, in management practice, leaders do not use merely a single type of leadership behavior; instead, they adopt a contingent leadership approach, dynamically adjusting strategies to drive the team. Theoretical studies indicate that transformational and transactional leadership are not polar opposites; leaders can possess both simultaneously, albeit to varying degrees and in different combinations. However, these combinations are not randomly infinite but are likely to form several representative and internally consistent leadership “patterns” or “types.” As a person‐centered analysis method, LPA can regard leaders as an integrated whole and identify different subgroups that are internally homogeneous and exhibit similar patterns in transformational and transactional leadership behaviors. In recent years, the field of organizational behavior research has increasingly called for the use of person‐centered methods to more comprehensively understand complex individual differences. The application of LPA to ambidextrous leadership research contributes to deepening our comprehension of leadership configurations and their efficacy.

Based on the literature review and the research objectives outlined above, we propose the following hypotheses:

**Hypothesis 1.**

(H1): There are significant associations between ambidextrous leadership, LMX, and nurses’ presenteeism.

**Hypothesis 2.**

(H2): The heterogeneity of ambidextrous leadership can be identified through LPA.

**Hypothesis 3.**

(H3): LMX mediates the relationship between ambidextrous leadership and nurses’ presenteeism.

## 2. Materials and Methods

### 2.1. Study Design

A multicenter cross‐sectional survey design was used.

### 2.2. Participants

From December 2024 to January 2025, 513 clinical nurses from three tertiary general hospitals in Guangzhou were selected for a questionnaire survey through convenience sampling. Inclusion criteria included the following: (1) Possession of a valid nursing license, (2) more than 1 year of work experience, and (3) voluntarily consenting to participate. Exclusion criteria included the following: (1) Nurses who go out for further training, (2) on long‐term leave (leave of absence of three months or more, e.g., sick leave or maternity leave), and (3) nurses from other hospitals in training and internship nurses.

### 2.3. Sample Size

The sample size required for this study was calculated using G∗Power 3.1 software. The effect size value was determined based on Ferguson’s established criteria for social sciences [26], in conjunction with findings from prior relevant research [27]. Given an effect size (f) of 0.25, an alpha level of 0.05, and a desired statistical power (1 − β) of 0.80, the analysis indicated that a total of 200 participants would be necessary. However, according to the sample size rule of thumb for latent profiling [28], a total sample size of at least 300 was required to ensure model stability. We distributed a total of 570 questionnaires. After recovery and the removal of 57 invalid questionnaires, we finally obtained 513 valid samples for analysis, with an effective recovery rate of 90%.

### 2.4. Measures

#### 2.4.1. Sociodemographic Characteristics

This part was designed by the researcher and included age, gender, education, working years, marital status, number of children, job position, whether night shift, sleep time, and health status.

#### 2.4.2. Ambidextrous Leadership Scale

The assessment of transformational leadership was based on the eight‐item Transformational Leadership Scale (TLS) designed by Yongxia Chen for the Chinese context, which covers the dimensions of charisma (*n* = 4 items) and inspirational power (*n* = 4 items) [29]. The reliability of the original scale, as assessed by Cronbach’s alpha, was 0.81. Items were rated on a five‐point Likert scale ranging from 1 (*strongly disagree*) to 5 (*strongly agree*). In this study, Cronbach’s α was 0.857, and the confirmatory factor analysis fits well (*χ* ^2^/*df* = 2.038, CFI = 0.985, TLI = 0.978, RMSEA = 0.045, SRMR = 0.024).

For the assessment of transactional leadership, the Multifactor Leadership Questionnaire (MLQ) developed by Avolio and Bass [16] was used, with 6 items containing 2 dimensions: discretionary rewards (*n* = 3 items) and management by exception (*n* = 3 items). Items were rated on a five‐point Likert scale ranging from 1 (*strongly disagree*) to 5 (*strongly agree*). In this study, Cronbach’s α was 0.853, and the confirmatory factor analysis fits well (*χ* ^2^/*df* = 2.245, CFI = 0.991, TLI = 0.984, RMSEA = 0.049, SRMR = 0.018).

#### 2.4.3. LMX Scale

The LMX Scale developed by Graen and Uhl‐Bien was used to assess nurses’ perceived leadership support and trust [23]. The scale consists of 7 items, including questions such as “I know how to support my leader” and “I have a good working relationship with my leader.” It is rated from 1 to 5 (1 = strongly disagree and 5 = strongly agree). In this study, Cronbach’s α was 0.844, and the confirmatory factor analysis fits well (*χ* ^2^/*df* = 2.706, CFI = 0.984, TLI = 0.972, RMSEA = 0.058, SRMR = 0.025).

#### 2.4.4. Stanford Presenteeism Scale

The scale was developed by scholar Koopman et al. [30] and translated by Chinese scholar Zhao et al. [31]. It is used to assess the decline in work efficiency (e.g., “I’m on the job but I have trouble concentrating”) in the past month due to health problems. The scale consists of 6 items and is divided into two main dimensions: work limitations (Items 1–4) and work energy (Items 5‐6), with work energy being reverse‐scored. Items were rated on a five‐point Likert scale ranging from 1 (*strongly disagree)* to 5 (*strongly agree*). The scale ranges from 6 to 30 points, and a higher score indicates a more serious decline in productivity caused by presenteeism. The presenteeism scores were divided into two groups, high presenteeism and low presenteeism, using the median as the cutoff point. In this study, Cronbach’s α was 0.934, and the confirmatory factor analysis fits well (*χ* ^2^/df = 2.862, CFI = 0.994, TLI = 0.989, RMSEA = 0.060, SRMR = 0.011).

### 2.5. Data Collection

This study was conducted via the Wenjuanxing platform on WeChat. With the approval of the nursing department, questionnaire links were distributed to eligible nurses. The research strictly adhered to principles of informed consent and privacy protection, with all responses collected anonymously. To ensure data robustness, uniform instructions were provided to the participating nurses, and submissions were restricted to one response per device or IP address. In addition, we included reverse‐scored questions in the questionnaire to eliminate logically inconsistent data, excluded responses completed in an excessively short time (≤ 120 s), and discarded data where the same option was selected for all questions. Finally, all collected data underwent rigorous manual review to eliminate records with abnormal IP addresses, thereby safeguarding the validity and reliability of the dataset.

### 2.6. Ethical Considerations

The study was performed according to the relevant guidelines and regulations and was approved by the Ethics Committee of The First People’s Hospital of Guangzhou (approval number: K‐2024‐149‐01; approval date: December 10, 2024). The study complied with the ethical standards of the committee responsible for human experimentation and the Helsinki Declaration.

### 2.7. Data Analysis

SPSS 27.0 and Mplus 8.3 were used in this study for statistical analysis of the collected data.

First, Harman’s univariate model was employed to assess the potential common method variance (CMV) issue [32].

Second, an LPA based on TLS and MLQ scores was conducted to identify subgroups reflecting different levels of ambidextrous leadership. Model fit was assessed using several indices: for Akaike information criterion (AIC), Bayesian information criterion (BIC), and adjusted BIC (aBIC), a smaller value represents a better model fit; the entropy index ranges from 0 to 1, with values closer to 1 representing higher classification accuracy; for the Lo–Mendell–Rubin test (LMRT) and bootstrap likelihood ratio test (BLRT), when comparing the model fitting effect, a *p* value less than 0.05 indicates that the model fit of k categories is better than that of k – 1 categories [33].

The data were further analyzed using SPSS 27.0. Categorical data were described using frequencies and percentages, while continuous data were described as mean ± standard deviation (SD). Between‐group comparisons were conducted using independent sample *t*‐tests or analysis of variance (ANOVA). When ANOVA indicated statistically significant overall differences, post hoc pairwise comparisons were performed. Pearson correlation analysis was used to examine relationships between variables. Subsequently, the bias‐corrected bootstrap with a Huber–White estimator (BCH) method was used to examine LMX and nurses’ presenteeism as distal outcomes of ambidextrous leadership profiles.

Finally, the mediating effect of LMX was examined using the macro program PROCESS 4.2. This study employed Model 4 of the PROCESS macro to examine the mediating effect of a single mediator (M) in the relationship between the independent variable (X) and the dependent variable (Y). The bootstrap sample size was set to 5,000, and a 95% bias‐corrected confidence interval (CI) was used. A significant mediation effect is indicated when the 95% bias‐corrected CI does not include zero.

## 3. Results

### 3.1. Common Method Bias Test

This study employed Harman’s single‐factor test to examine whether common method bias significantly influenced the results. An exploratory factor analysis was conducted, including all 27 measurement items. The unrotated factor solution extracted five factors with eigenvalues over 1, with the largest factor accounting for 30.502% of the variance, which is below the critical threshold of 40% [34]. The sample data did not exhibit significant common method bias.

### 3.2. LPA of Ambidextrous Leadership

The 14‐item scores based on the TLS and MLQ were divided into 1 to 4 profile models, and the model fit metrics are shown in Table 1. With the increase in the number of profiles, the values of AIC, BIC, and aBIC decreased gradually. Among the four classification models, the LMRT and BLRT of the first three classes reached a significant level (*p* < 0.05). Class 3 had the smallest values of AIC, BIC, and aBIC, and its entropy value was 0.953, which was larger than that of Class 2. After comprehensively comparing the fit indexes of each class, Class 3 was selected as the best latent profile class. The attribution probability matrices of the three latent profiles showed that the average attribution probabilities of each profile were 0.994, 0.947, and 0.981, respectively, indicating that the model classification results were reliable. We calculated the mean scores of the three profiles of ambidextrous leadership across the 14 items. Items A1–A8 were from the TLS, and Items B1–B6 were from the MLQ. We named C1∼C3 based on each latent profile’s score on each item. The C1 profile of ambidextrous leadership had low mean scores on both the TLS and MLQ dimensions, so it was named “Ambidextrous Lagging,” which accounted for 40.9% of nurses. The C2 profile had high mean scores on the TLS dimension but low mean scores on the MLQ dimension, so it was named “Vision‐Oriented,” which accounted for 17.9% of nurses. The C3 profile had high mean scores on both the TLS and MLQ dimensions, so it was named “Ambidextrous Synergistic,” which accounted for 41.1% of nurses. The latent profile plot can be seen in Supporting Figure 1.

**TABLE 1:** Fitting index of the latent profile model of ambidextrous leadership.

Model	AIC	BIC	aBIC	Entropy	LMRT	BLRT	Class probability
1	15611.955	15730.683	15641.806	—	—	—	—
2	13339.946	13522.278	13385.789	0.946	< 0.001	< 0.001	0.452/0.548
**3**	**12820.297**	**13066.233**	**12882.131**	**0.953**	**<** **0.001**	**<** **0.001**	**0.409/0.179/0.411**
4	12605.175	12915.715	12683.001	0.962	0 .19	< 0.001	0.043/0.172/0.385/0.399

*Note:* Bold values indicate the selected optimal model based on model fit indices and statistical tests.

### 3.3. Comparison of the General Data of Clinical Nurses in Different Ambidextrous Leadership

The results showed that the majority of the 513 respondents, 494 in total, were women; 49.5% of the nurses were older than 35 years old (*n* = 254); 60.6% of the nurses had been working for more than 10 years (*n* = 311). As shown in Table 2, a comparison of the three latent profiles of ambidextrous leadership in terms of nurses’ age, work experience, marital status, number of children, and health status showed statistically significant differences (*p* < 0.05).

**TABLE 2:** Comparison of the general data of clinical nurses in different ambidextrous leadership (*N* = 513).

Variables	Classification	C1	C2	C3	*χ* ^2^/H	*p*
Age (years) (*n*%)	< 25	43 (58.9)	11 (15.1)	19 (26.0)	17.780	**0.023**
	25∼30	42 (46.7)	18 (20.0)	30 (33.3)		
	31∼35	32 (33.3)	17 (17.7)	47 (49.0)		
	36∼40	43 (37.4)	19 (16.5)	53 (46.1)		
	> 40	50 (36.0)	27 (19.4)	62 (44.6)		

Gender (*n*%)	Male	12 (63.2)	2 (10.5)	5 (26.3)	4.037	0.133
	Female	198 (40.1)	90 (18.2)	206 (41.7)		

Education (*n*%)	College and below	39 (54.7)	10 (14.1)	22 (31.0)	8.950	0.062
	Undergraduate	164 (39.0)	80 (19.0)	177 (42.0)		
	Postgraduate and above	7 (33.3)	2 (9.5)	12 (57.1)		

Working years (*n*%)	0∼5	72 (55.4)	20 (15.4)	38 (29.2)	21.502	**0.001**
	6∼10	33 (45.8)	15 (20.8)	24 (33.3)		
	11∼15	38 (31.7)	21 (17.5)	61 (50.8)		
	> 15	67 (35.1)	36 (18.8)	88 (46.1)		

Marital status (*n*%)	Unmarried	88 (52.1)	29 (17.2)	52 (30.8)	14.867	**0.005**
	Married	119 (35.2)	62 (18.3)	157 (46.4)		
	Divorce	3 (50)	1 (16.7)	2 (33.3)		

Children (*n*%)	0	95 (49.5)	33 (17.2)	64 (33.3)	13.105	**0.011**
	1	54 (32.3)	36 (21.6)	77 (46.1)		
	≥ 2	61 (39.6)	23 (14.9)	70 (45.5)		

Position (*n*%)	Nurse	138 (45.2)	53 (17.4)	114 (37.4)	8.640	0.071
	Head of nursing team	41 (39.0)	16 (15.2)	48 (45.7)		
	Head nurse and above	31 (30.1)	23 (22.3)	49 (47.6)		

Night shifts (*n*%)	Yes	165 (44.0)	63 (16.8)	147 (39.2)	5.460	0.065
	No	45 (32.6)	29 (21.0)	64 (46.4)		

Sleep duration (h/d) (*n*%)	≤ 6	102 (41.5)	46 (18.7)	98 (39.8)	0.379	0.827
	> 6 h	108 (40.4)	46 (17.2)	113 (42.3)		

Health status (*n*%)	Very poor	8 (61.5)	3 (23.1)	2 (15.4)	24.765	**0.002**
	Below average	35 (55.6)	7 (11.1)	21 (33.3)		
	Average	135 (43.0)	55 (17.5)	124 (39.5)		
	Above average	30 (27.0)	26 (23.4)	55 (49.5)		
	Excellent	2 (16.7)	1 (8.3)	9 (75.0)		

*Note:* C1 = ambidextrous lagging; C2 = vision‐oriented; C3 = ambidextrous synergistic. *χ* ^2^ = chi‐square test; H = Kruskal–Wallis test. Bold values indicate *p* < 0.05.

### 3.4. Differences in LMX and Nurses’ Presenteeism Across Latent Profiles of Ambidextrous Leadership

In this study, a one‐way ANOVA test was used to compare the different latent profiles of ambidextrous leadership in terms of LMX and nurses’ presenteeism. The results showed significant differences among the latent profiles for both LMX (*F* = 58.986, *p* < 0.001) and nurses’ presenteeism (*F* = 15.482, *p* < 0.001). Post hoc comparisons (LSD) revealed that ambidextrous synergistic scored significantly higher on LMX than ambidextrous lagging and vision‐oriented. Ambidextrous lagging scored significantly higher on nurses’ presenteeism than vision‐oriented and ambidextrous synergistic, but there was no difference between vision‐oriented and ambidextrous synergistic (Table 3).

**TABLE 3:** Differences in LMX and nurses’ presenteeism across latent profiles of ambidextrous leadership (mean ± SD).

	Ambidextrous lagging (I)	Vision‐oriented (II)	Ambidextrous synergistic (III)	*F*	*η* ^2^	Post hoc (LSD)
LMX	27.45 ± 3.59	29.33 ± 3.13	30.78 ± 2.65	58.986^∗∗∗^	0.188	I < II < III
Presenteeism	15.67 ± 3.97	13.63 ± 4.01	13.26 ± 5.44	15.482^∗∗∗^	0.057	III, II < I

*Note: F* = *F*‐statistic; LMX, leader–member exchange.

Abbreviations: LLCI, lower level of confidence interval; LSD, least significant difference; SE, standard error; ULCI, upper level of confidence interval.

^∗∗∗^ *p* < 0.001.

### 3.5. BCH Approach

Given the uncertainty in latent class membership (entropy = 0.953), we further employed the BCH method to correct classification errors. Therefore, the BCH approach was used for LMX and nurses’ presenteeism, which served as association variables for the co‐occurrence of ambidextrous leadership profiles. The results show that there are significant differences in the impact of each ambidextrous leadership profile on LMX (*χ* ^2^ = 118.344, *p* < 0.001) and nurses’ presenteeism (*χ* ^2^ = 33.741, *p* < 0.001). The mean differences in LMX and nurses’ presenteeism across the latent profiles showed that the score for LMX was highest in the ambidextrous synergistic profile. The order of LMX scores across the three profiles was as follows: ambidextrous lagging < vision‐oriented < ambidextrous synergistic, suggesting that individuals who perceive effective leadership may experience higher‐quality leader–member relationships. In contrast, for nurses’ presenteeism, the ambidextrous lagging profile had the highest score. The order of nurses’ presenteeism scores was as follows: ambidextrous synergistic < vision‐oriented < ambidextrous lagging, indicating that individuals who perceive a lack of leadership support in their work may exhibit more frequent presenteeism behavior (see Supporting Table 1).

### 3.6. The Mediation Effect Analysis of LMX

Through LPA of ambidextrous leadership, three distinct profiles were identified. The LPA classification results were converted into dummy variables for subsequent regression and mediation analyses, using the ambidextrous lagging profile as the reference category. We used the SPSS macro program PROCESS to conduct a mediation analysis, with ambidextrous leadership as the independent variable and nurses’ presenteeism as the dependent variable. After controlling for age, work experience, marital status, number of children, and health status, we employed the bootstrap method to sample 5,000 times to test the mediating effects. The results showed that the total effect of the overall mediation analysis was significant, *F* (2, 505) = 9.434, *p* < 0.001. The overall direct effect was also significant, *F* (2, 504) = 1.345, *p* < 0.001. The 95% bootstrap CI of the overall mediation effect test was [−0.1041, −0.0434]. Since this interval did not include 0, further relative mediation analysis was necessary.

**FIGURE 1:**
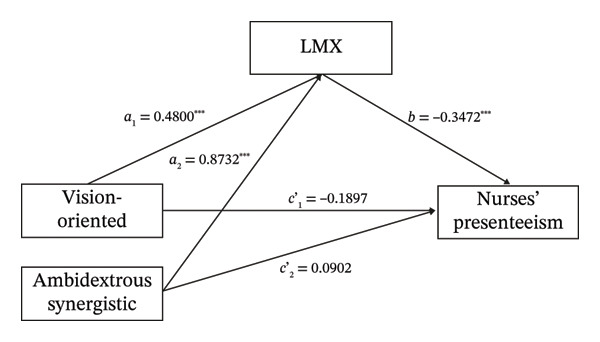
A hypothesized mediator model with three LPA‐based ambidextrous leadership as an independent variable (X), LMX as a mediator (M), and nurses’ presenteeism as a dependent variable (Y). *Notes.* ^∗∗∗^ *p* < 0.001. Ambidextrous lagging is used as the reference. Age, work experience, marital status, number of children, and health status are used as control variables, and the control variables are not presented in the figure for brevity.

Relative mediation analysis results showed that, using ambidextrous lagging as the reference, the 95% bootstrap CI of the relative mediation of vision‐oriented compared to ambidextrous lagging was [−1.2046, −0.4121]. Since this interval did not include 0, it suggested that the relative mediation effect was significant (*a*_1_ = 0.4800, *b* = −0.3472, *a*_1_ *b* = −0.1667). That is, the LMX of nurses in the vision‐oriented profile was 0.48 times higher than that of nurses in the ambidextrous lagging profile (*a*_1_ = 0.4800), and the nurses’ presenteeism in the vision‐oriented profile correspondingly decreased (*b* = −0.3472). The relative direct effect was not significant (*c*’_1_ = −0.8959, *p* = 0.1042), indicating that after introducing the mediating variable, the vision‐oriented profile has no direct impact on nurses’ presenteeism. The relative total effect was significant (*c*_1_ = −1.6905, *p* = 0.003), and the effect size of the relative mediation effect *a*_1_ *b* was 53%. Similarly, the 95% CI of the relative mediation of ambidextrous synergistic compared to ambidextrous lagging was [‐1.8854, −1.0455], which did not include 0, indicating that the relative mediation effect was significant (*a*_2_ = 0.8732, *b* = −0.3472, *a*_2_ *b* = −0.3031). That is, the LMX of nurses in the ambidextrous synergistic profile was 0.8732 times higher than that in the ambidextrous lagging profile (*a*_2_ = 0.8732), and their presenteeism correspondingly decreased (*b* = −0.3472). The relative direct effect was not significant (*c*’_1_ = −0.4299, *p* = 0.3600), indicating that after introducing the mediating variable, the ambidextrous synergistic profile has no direct impact on nurses’ presenteeism. The relative total effect was significant (*c*_2_ = −1.8754, *p* < 0.001), and the effect size of the relative mediation effect *a*_2_ *b* was 16.17% (Tables 4 and 5 and Figure 1).

**TABLE 4:** Tests of the mediating effect of LMX in the relationship between ambidextrous leadership and nurses’ presenteeism.

Outcome variables	Independent variables	Unadjusted analysis							Adjusted analysis						
		*β*	SE	*t*	*p*	LLCI	ULCI	*R* ^2^	*β*	SE	*t*	*p*	LLCI	ULCI	*R* ^2^
LMX	Vision‐oriented	0.5386	0.3938	4.7706	< 0.001	1.1049	2.6521	0.1879	0.4800	0.3934	4.2556	< 0.001	0.9012	2.4470	0.2194
	Ambidextrous synergistic	0.9546	0.3070	10.8461	< 0.001	2.7265	3.9328		0.8732	0.3132	9.7227	< 0.001	2.4301	3.6609	

Nurses’ presenteeism	Vision‐oriented	−0.2240	0.5554	−1.9266	0.0551	−2.1588	0.0234	0.1739	−0.1879	0.5505	−1.6275	0.1042	−1.9774	0.1856	
	Ambidextrous synergistic	−0.1448	0.4699	−1.4690	0.1425	−1.6136	0.2329		−0.0902	0.4692	−0.9163	0.3600	−1.3518	0.4920	0.2118
	LMX	−0.3790	0.0611	−8.4792	< 0.001	−0.6382	−0.3981		−0.3472	0.0612	−7.7577	< 0.001	−0.5948	−0.3544	

*Note:* Unadjusted analysis: nonadjusted controlled variables. Adjusted analysis: age, work experience, marital status, number of children, and health status as control variables. “Ambidextrous lagging” as the reference.

**TABLE 5:** Direct and indirect effects of ambidextrous leadership (categorical variables) on nurses’ presenteeism.

Effects	Variables	Unadjusted analysis				Adjusted analysis			
		Estimate	SE	LLCI	ULCI	Estimate	SE	LLCI	ULCI
Indirect effect	Vision‐oriented	−0.9733	0.2246	−1.4258	−0.5358	−0.7946	0.2043	−1.2046	−0.4121
	Ambidextrous synergistic	−1.7251	0.2236	−2.1779	−1.3065	−1.4455	0.2149	−1.8854	−1.0455

Direct effect	Vision‐oriented	−1.0677	0.5554	−2.1588	0.0234	−0.8959	0.5505	0.1042	−1.9774
	Ambidextrous synergistic	−0.6904	0.4699	−1.6136	0.2329	−0.4299	0.4692	0.3600	−1.3518

Total effect	Vision‐oriented	−2.0410	0.5799	−3.1803	−0.9017	−1.6905	0.5717	−2.8136	−0.5673
	Ambidextrous synergistic	−2.4155	0.4521	−3.3037	−1.5273	−1.8754	0.4522	−2.7697	−0.9811

*Note:* Unadjusted analysis: nonadjusted controlled variables. Adjusted analysis: age, work experience, marital status, number of children, and health status as controlled variables. “Ambidextrous lagging” as the reference.

Abbreviations: LLCI, lower level of confidence interval; SE, standard error; ULCI, upper level of confidence interval.

## 4. Discussion

This study is the pioneer in utilizing LPA to identify different ambidextrous leadership patterns in the field of nursing management. In addition, ambidextrous leadership was found to have an indirect effect on nurses’ presenteeism, mediated by LMX. These findings highlight the influence of the external environment (e.g., ambidextrous leadership) and individual cognition and emotion (e.g., LMX) on behavioral attitudes (e.g., presenteeism).

The results showed that 41.1% of nurses perceived their leaders as ambidextrous synergistic, and had the lowest presenteeism scores (13.26 ± 5.44), indicating that this type of leader flexibly switches leadership styles in different situations, balances innovation and efficiency, and motivates nurses’ professional identity through visions and uses norms to clarify nurses’ responsibilities. This conclusion confirms BASS’s [16] view that both transformational and transactional leadership can complement and coexist while acting on employee performance. Cao’s [35] study showed that ambidextrous leadership can improve the psychological resilience of medical staff and enhance satisfaction and well‐being, thus soothing work fatigue. In addition, the study also showed that ambidextrous leadership can simultaneously influence employees’ innovative behavior through cognition and emotion [36]. Ambidextrous synergistic leadership focuses on the complementary strengths and weaknesses of transformational and transactional leadership, where the leader positively influences the behavioral attitudes of nurses in terms of both emotions and task goals [25]. Transformational leadership transforms the negative emotions of nurses through emotional empowerment, gives nurses the psychological implication of “I can do it” and “I am capable of meeting challenges” [37, 38], and re‐interprets the value of nurses in their work tasks, thus stabilizing their core and strengthening their beliefs [39]. Transactional leadership emphasizes task performance and reward and punishment mechanisms. This ensures nurses’ work efficiency, task completion, and sense of responsibility at work, which enhances nurses’ job satisfaction and reduces presenteeism, thereby avoiding damage to organizational productivity [40, 41]. Notably, 40.9% of nurses perceived their managers’ leadership as ambidextrous lagging, a category reflecting lower leadership competency. Consequently, this group also recorded the highest presenteeism scores (15.67 ± 3.97). The low level of leadership effectiveness of this type of manager may lead to multiple organizational risks. At the individual level, it may cause increased professional confusion and decreased job satisfaction among nurses. At the team level, it may weaken cohesion and increase talent loss. Ultimately, work efficiency may decline sharply, making it difficult for the organization to achieve long‐term sustainable development [42, 43].

This study found significant demographic differences in perceived leadership styles among the nurse population. The ambidextrous synergistic leadership is more commonly seen in nurses who are older, highly educated, senior, and married with children. Young nurses had a higher perception of vision‐oriented leadership, which may be attributed to the demands of the new generation of medical staff for career development. Young nurses are mostly in the period of career exploration and have a strong demand for professional competence enhancement and career development opportunities [44]. However, due to the short time new nurses have been in the workplace, they are often confused due to insufficient clinical skills, ambiguous role positioning, or a low sense of career awareness [45], and vision‐oriented leaders can precisely meet their developmental demands by constructing a clear blueprint for career development (e.g., pathways of training for specialized nurses and opportunities for research participation) [46]. The ambidextrous lagging leadership pattern, which is most often seen in subhealthy nurses, suggests that inadequate leadership management effectiveness may exacerbate the vicious cycle of occupational health problems [47], i.e., when subhealthy groups that should be supported by the organization are continually exposed to ineffective leadership environments, the process of recovery of their psychophysiological resources will be further retarded.

According to the hypothesis, an analysis based on the human‐centered mediation model indicates that LMX plays a significant mediating role between ambidextrous leadership and nurses’ presenteeism. Specifically, the ambidextrous synergistic group demonstrated higher‐quality LMX, while nurses exhibited fewer presenteeism behaviors. The study suggests that positive and effective leadership can enhance the exchange relationship between leaders and nurses, boost nurses’ sense of belonging and trust in their leaders, and improve their emotional and psychological well‐being, thus influencing their work behaviors [48, 49]. Conversely, toxic leadership can lead to emotional exhaustion among nurses, weakening the exchange relationship with leaders and potentially triggering defensive or counterproductive behaviors [50]. Therefore, given its dual impact on individuals and organizations, nursing managers need to adopt more effective interventions. According to SET, the ambidextrous synergistic leadership not only conveys emotional commitment to nurses through personalized care but also utilizes fair reward–punishment systems and clear rules to help nurses understand their roles, thereby enhancing their psychological safety. The resources and encouragement from leaders can motivate nurses to engage in positive behaviors, prompting ambidextrous synergistic leaders to provide more support [51]. Through such continuous social exchange and positive interactions, both parties can establish and maintain high‐quality exchange relationships. Thus, ambidextrous synergistic leaders are more likely to maintain high levels of LMX with nurses. Nurses with high LMX actively reduce presenteeism behaviors to avoid harming organizational interests [52].

This study outlines management strategies to protect nursing staff in healthcare environments, emphasizing the crucial role of awareness leadership styles in nurse development, and tailored interventions are recommended based on leadership profiles. For the ambidextrous lagging leadership profile: (1) implement a leadership reinvention program: conduct multidimensional leadership assessments to identify managers’ competency gaps in integrating transformational and transactional leadership styles. (2) Modular training curriculum: Allow managers to customize learning modules to address specific leadership deficiencies. (3) Compensatory resources for nurses: Provide psychological counseling to mitigate emotional exhaustion caused by inconsistent leadership, thereby enhancing nurses’ psychological resilience. For the vision‐oriented leadership profile, balance demands with incentives: Excessive performance expectations without tangible rewards may escalate nurses’ presenteeism, and complement visionary leadership with a structured incentive system, such as career advancement opportunities tied to goal achievement, and performance‐based salary increments.

### 4.1. Limitations

This study proposes a pioneering theoretical model that links ambidextrous leadership, LMX, and presenteeism in nurses. However, several limitations should be noted. First, this study employed a cross‐sectional design, which makes it difficult to clearly determine the causal temporal sequence among variables. There may be issues of reverse causality, where the status of the mediator or dependent variable may affect the independent variable, thereby interfering with the accurate identification of the mediating path. Moreover, although some demographic and work‐related variables were controlled for, there may still be other unmeasured confounding factors that could bias the estimation of the mediating effect. In addition, the effect may have temporal lags. Future research could employ longitudinal studies or randomized controlled trials (RCTs) to provide temporal support for the causal relationships between variables. The cross‐lagged model could be utilized to examine the bidirectional relationships between variables in longitudinal data collected at multiple time points, clarifying the direction of effects and their temporal lag characteristics.

Second, the data were solely based on nurse self‐reports, which may introduce subjectivity and CMV, potentially limiting the generalizability of the findings. Moreover, the sample was self‐selected and recruited online, so those nurses with higher levels of digital engagement or stronger opinions on the study topic may be overrepresented. Therefore, the findings may not be applicable to the broader population of nurses. For future studies, it is advisable to adopt probability‐based panels or multistage stratified sampling with offline recruitment.

Third, the use of convenience sampling, which only included nurses from three tertiary hospitals in Guangzhou, China, resulted in selection bias. This limited the representativeness of the sample, making it difficult to generalize the study findings. To mitigate selection bias, it is recommended to prioritize probability sampling methods (such as simple random sampling, stratified sampling, or cluster sampling) for subject selection. In addition, to enhance the study’s external validity, future research should incorporate nursing samples from diverse geographic regions and cultural backgrounds.

## 5. Conclusion

This paper contributes new knowledge regarding ambidextrous leadership and presenteeism, which is a domain that has been neglected in previous health services research. This study demonstrated a negative correlation between ambidextrous leadership and presenteeism among nurses, with LMX acting as a mediating variable in this relationship. Furthermore, LPA revealed heterogeneity in ambidextrous leadership and had a differentially affected nurses’ presenteeism. The results of the study suggest that we must address the issue not only from the nurses’ perspective but also from the perspective of the nursing administrators to address the impact of leadership on nurses’ presenteeism.

## Author Contributions

Xiuran Zhou: conceptualization, methodology, validation, formal analysis, data curation, and writing–original draft. Ying Yu: data curation, validation, and writing–review and editing. Xiaoying Yan: conceptualization and writing–review and editing.

## Funding

No funding was received for this research.

## Disclosure

The authors agree to take responsibility for ensuring that the choice of statistical approach is appropriate and is conducted and interpreted correctly as a condition to submit to the Journal.

## Conflicts of Interest

The authors declare no conflicts of interest.

## Supporting Information

Additional supporting information can be found online in the Supporting Information section.

## Supporting information


**Supporting Information 1** Figure S1. Latent profile subplots of ambidextrous leadership.

**Supporting Information 2** Table S1. Comparing the differences in LMX and nurses’ presenteeism across different latent profiles, M (SE).

## Data Availability

Research data are not shared.
