# Supplementary figures and images for: Assessing the Impact of Ambidextrous Leadership on Nurses’ Presenteeism: A Latent Profile and Mediation Analysis Study
Source: J Nurs Manag. 2026 Feb 27;2026:6624868. doi: 10.1155/jonm/6624868 (PMC12946810; doi:10.1155/jonm/6624868)

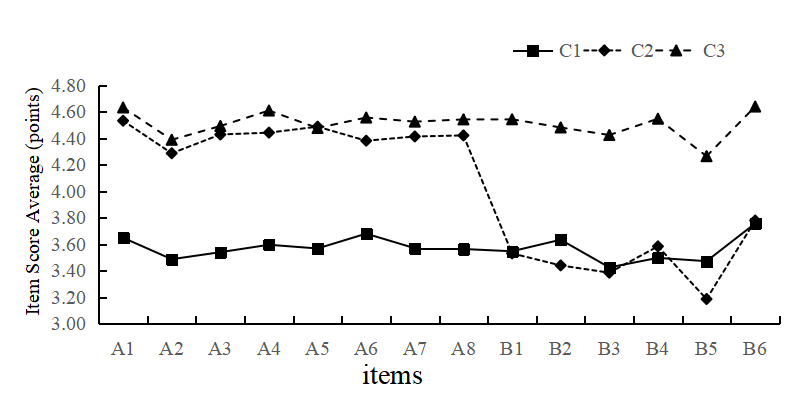


**Figure S1. Latent profile subplots of ambidextrous leadership**

Supplement: Supplementary file 1 — Supporting Information 1 Figure S1. Latent profile subplots of ambidextrous leadership. [file JONM-2026-6624868-s001.doc]
