# Supplementary material for: Assessing the Impact of Ambidextrous Leadership on Nurses’ Presenteeism: A Latent Profile and Mediation Analysis Study
Source: J Nurs Manag. 2026 Feb 27;2026:6624868. doi: 10.1155/jonm/6624868 (PMC12946810; doi:10.1155/jonm/6624868)
Supplement: Supplementary file 2 — Supporting Information 2 Table S1. Comparing the differences in LMX and nurses’ presenteeism across different latent profiles, M (SE). [file JONM-2026-6624868-s002.docx]

| **Table S1** Comparing the differences in leader-member exchange and nurses’ presenteeism across different latent profiles, M (SE) | | | | | |
| --- | --- | --- | --- | --- | --- |
| Variables | M (SE) | Pairwise comparisons(χ^2^) | | | Overall test (χ^2^) |
|  |  | C1 vs C2 | C1 vs C3 | C2 vs C3 |  |
| LMX | C1=27.43(0.25) | 19.29*** | 117.96*** | 14.39*** | 118.344*** |
|  | C2=29.31(0.34) |  |  |  |  |
|  | C3=30.82(0.19) |  |  |  |  |
| Nurses’ presenteeism | C1=15.69(0.28) | 15.87*** | 26.87*** | 0.38 | 33.741*** |
|  | C2=13.61(0.44) |  |  |  |  |
|  | C3=13.24(0.38) |  |  |  |  |
| *Notes.* C1 = ambidextrous lagging, C2 = vision-oriented, C3 = ambidextrous synergistic; ****P*＜0.001；LMX = leader-member exchange. | | | | | |
